# Remarkable Effects of Fanaticism on the Inhabitants of Several Towns in Cornwall

**Published:** 1814-05

**Authors:** James Cornish

**Affiliations:** Falmouth


*For the Medical and Physical Journal.*

### *Remarkable Effects of Fanaticism on the Inhabitants of several Towns in Cornwall.* By Mr. JAMES CORNISH.

A CONVULSIVE epidemic has recently made its appearance in Cornwall, which has extended its effects over a considerable part of the county, and to several thousands of individuals. This disease is remarkable for the manner in which it has been propagated, and for the singularity of many of its symptoms. Religious enthusiasm has been the exciting cause, and this has operated with a degree of violence of which no one but an eye-witness can form an adequate idea. It took its rise in a chapel belonging to that class of dissenters known by the name of Wesleyan methodists, in the town of Redruth. During the time of service, a man cried out loudly, to the astonishment of the congregation, "What shall I do to be saved?" and expressed a most alarming apprehension as to the state of his soul. Many persons, following his example, repeated his exclamation, and they in a short time appeared to labour under the greatest bodily pain.

The account of this singular proceeding was soon made public, and hundreds of individuals, prompted by curiosity or other motives to be spectators, became similarly affected through the medium of the principle of imitation.

The chapel in which it originated was kept open for several nights and days; and from thence it spread with the rapidity of lightning to the neighbouring towns and their adjacent villages.* As it approached these places, it subsided in some measure where it first appeared, but wherever it has been seen, it has been confined wholly to the chapels of the sect; it has originated from some one crying out in the manner above related, and it has prevailed chiefly amongst those whose intellect is of the very lowest class. In every place, those who were influenced by the example given them, expressed their agony by convulsive motions of the limbs, and many by crying out in the most frightful manner, that the anger of the Almighty was about to be poured out upon them, that they heard the shrieks of the tormented spirits, and that they saw hell opening to receive them. As soon as the ministers who preached to them observed them thus affected, they begged them to encourage their convictions, and laboured most earnestly to impress them with a belief that by nature they were the enemies of Christ, that therefore the wrath of God was upon them, and that if they died in their sins, their portion would be in everlasting flames. Those whom they thus addressed repeated the words, and immediately applied them to their own consciences. This operated, as might be imagined, to increase the fury of the convulsive paroxysm. When this belief had been thought well grounded in them, the preachers changed the nature of their discourses, and exhorted them to trust in the merits of an all-sufficient Saviour, to believe in the mercy of God, to pray for a supply of faith that would enable them to believe that their sins were forgiven, and then pictured to their hopes the joys of heaven in glowing colours. Thus according to the degree of faith they imagined that they had received was their conversion, or belief that their sins were forgiven, rapid. Some, however, laboured under the sense of conviction many days, but in by far the greater number of cases their conversion was brought about in a manner surprisingly sudden. In a moment they were elevated to the most exalted state of happiness from the lowest depth of misery and despair, and they exclaimed with joy and triumph that their chains were loosed, that their sins were forgiven, and that they were brought into the marvellous liberty of the sons of God. Notwithstanding this

* Camborne, Helston, Truro, Penryn, and Falmouth.

considerable considerable change of feeling in the mind, the convulsive motions often continued. The stimulus of the exciting cause has operated so powerfully that every secular object has been neglected, and numbers of the individuals who have been under its influence, have remained in the chapel where they first experienced it for two or three days and nights together, without sustenance and without rest, incessantly in convulsive motion. From the accounts I have received from the different parts of the county where this singular affection has prevailed, I suppose that not less than four thousand individuals have been subjected to its influence.

The following is a general outline of the progress of the attack. A sensation like syncope, and a coldness and sense of weight about the præcordia, are the symptoms first experienced; and soon after the patients shriek, as if in considerable agony. From the exclamations which escape many of the females, one would be almost led to conclude that they were in labour. In the first case I saw (a female), and to which I gave particular attention, I thought it was absolutely the case. The muscles of the eyes are first convulsed, but these organs soon become fixed and staring. The muscles of the face are next contorted in a most hideous manner. In a short time those of the throat and trunk partake of the commotion, and at every expiration the air is thrown out as if the patients were troubled with singultus: at the same time they shake and tremble, cry piteously, and toss the head from side to side with great agitation. As the disease gains strength it proceeds to the superior extremities; and now the patients beat their breasts, clasp their hands, and perform a great variety of muscular gesticulation. I have not observed the lower extremities to have been equally affected in any of the cases I have witnessed. When these parts are seized, the action is carried on with a violence truly astonishing, and it continues an hour, two hours, or longer, according to the degree of strength of the convulsed person. In some cases the nervous energy is exhausted in a few minutes, but the fit is usually of much longer duration. It has even been protracted to seventy and eighty hours. Many of the subjects who were seated during the paroxysm rapidly bent their bodies forwards and backwards with corresponding motions of the arms similar to a man in the act of sawing timber. Others shouted, jumped, and writhed themselves into every posture of which their limbs were susceptible, and these they continued until their strength was exhausted. Yawning is at first a constant symptom, indicating dicating accumulation in the cavities of the heart;* but as the distemper gathers force, the extreme agitation of the body is such, that the circulation becomes hurried, the breathing quickened, and the countenance swollen and bloated. When the strength is nearly exhausted, the patients usually swoon, and they remain in a rigid and motionless state, until respite from fatigue, fresh air, or some other more powerful stimulus, enables them to re-act, or to put a stop to the curious character they had undertaken. From attentive observation I have been led to conclude that the persons affected are in a state of perfect consciousness during the paroxysm. In the incipient stage it very much resembles chorea, but towards the close its violence is so much increased that I have seen a female effectually resist the efforts of four or five robust men to hold her. Any attempt to restrain them during the exacerbation of the paroxysm only made them doubly furious: they were therefore for the most part permitted to exercise themselves freely, until nature was no longer able to support the action. After the fit they complain of fatigue and soreness, and these are proportionate to the violence of the muscular action. The affection is not confined to sex, or age, or particular constitution. Women afford more instances of it than men. Children of five or six, and old men of eighty, have been attacked; but its principal range is among girls and young women. Robust constitution is not exempt from an attack. Its influence, however, has been chiefly exerted on persons whose minds are of the very lowest order, and particularly on those who are disposed to be enthusiasts in religion. Inability to continue the action is the only remission the disease experiences. In one case it is said to have caused phrenitis, and in several others a degree of melancholy, remote however from fear or despair, as they have assured me they feared neither death nor hell. I have not known that it has proved mortal in a single instance. The state of the weather during the time of its first appearance was fair; the air dry and cold.

As to the exciting cause of this disease, a variety of opinions exists. Some ascribe it to the spirit of God, others to the spirit of the Devil, others to intoxication, others to insanity, and others to the influence of the passions.

The sect with whom this singular affection originated, holding an opinion that the spirit of God operates sensibly

* "The heart, when affected by any extraordinary impression, collapses, so as neither to receive nor emit any blood."—*Zimmermann on the Passions considered as a remote cause of Disease.*

in in the conversion of men, ascribe this work to divine agency; and in proof of this opinion adduce as an analogous instance the conversion of three thousand men, under the influence of a sermon preached them by the apostle Peter. When to this is opposed the improbability that the Divine Spirit should confine its operations to the chapels of their particular sect, they reply, that "God works how and where he pleases." The firmness of persuasion of the converts that the influence is divine, is also regarded by them as additional proof of its reality. But if strength of persuasion be the light that must guide us, how shall any one distinguish between the delusions of Satan and the inspirations of the Holy Ghost? If we allow their strong belief to judge the case, it will decide for their opinion; but firm persuasion, without any other proof, can never be admitted as sufficient evidence that any work is from God.*

To those who have ascribed it to the Devil, an objection is urged by the retainers of the first opinion, which, if founded on fact, will be admitted forcible, viz. that the Devil would not work on the minds of men to induce them to renounce the pleasures arising from a life of dissipation, and to act in a manner which would defeat his purpose. Notwithstanding, however, the diffused influence of the cause, and the affirmation of the opposing party as to its tendency, the aggregate of evil in these parts sustains a very inconsiderable diminution.

Others maintain that the abuse of spirituous liquors is the exciting cause, and, as far as their observation extended, this might have been the case: but it is inconclusive to draw a general inference from a solitary fact. Besides, the cause alleged produces different effects on different constitutions; but in the affection under consideration, the effects, though differing in degree, were in their nature similar wherever it prevailed.

Insanity is what has been also held adequate to explain the affection; but the effects were too diffused, and bore too general a resemblance to warrant the conclusion.

Those who have considered the subject seriously and without prejudice, think that there is no necessity to have recourse either to divine or to diabolical interference to explain it, but assert that natural causes are amply adequate to account for all its effects. The convulsive motions, they say, may be explained from the sympathy which is well known to exist between the body and soul. The influence

* Locke on Enthusiasm.

of the passions on the human frame is truly astonishing. They operate sometimes suddenly, at others slowly and imperceptibly, but their effects are equally certain. The particular mode in which they act on the human constitution has not been determined, but nevertheless it is clearly understood that a very intimate connexion subsists between the body and the mind, for what injures the one disorders the other. Sterne humorously compares them to a coat and its lining; if you rumple the one you rumple the other. Fear is the passion which of all others produces the most sensible effects. Zimmermann says, the usual effect of terror is to occasion epilepsy. At Gottingen he saw a woman attacked with epilepsy, from being suspected of having killed her child; and Wepfer relates a case where a patient became epileptic from sudden fright, and then died of apoplexy. Fear was the passion to which the sermons and exhortations of the ministers, in the convulsive affection above described, were principally directed, and that in a manner truly terrific. Hell, everlasting punishment, damnation, and all its tortures, were exposed to their view; the misery of which they would partake if they died in their sins, was painted in the fiercest coloring, and every circumstance that an ardent fancy could suggest, was added to heighten the horror of the scene. These were incessantly repeated in terms so awful and alarming, that when the imbecility and ignorance of the convulsed persons are duly considered, it can excite no astonishment that corresponding emotions should have been produced in their minds. When the strong sympathy which exists between the soul and body is also properly estimated, it must appear perfectly natural, that a violent passion of the one should produce a corresponding violent action of the other. This will also account for the change of feeling experienced when objects of a different nature were held up to them. After the disease had been once established, its propagation, they maintain, was effected through the medium of the imitative principle. Aristotle defines man an imitative animal; and so true is this, that we are not only disposed to imitate the pleasurable sensations of others, but also their painful sensations. The propensity to become affected with epilepsy from seeing any one in a fit, was well known to the Romans. This led them to forbid an affected person from appearing in the Comitia. From its having occurred in this place, and from its having been propagated to those who crowded round the person in the fit, they called it *Morbus Comitialis.* In the hospital at Haerlem, almost all the boys and girls were seized with convulsions convulsions from seeing a girl who had been frightened into them. That women are often seized with hysteric fits from seeing others attacked with them, is known to every one. Many instances have occurred in this epidemic of children, who rivalling adults in their violence, when asked the reason of their noise and agitation, replied they could not help it—they must do as the rest did. The plan which Boerhaave advised to be adopted in the hospital at Haerlem, of burning the patient in the arm to the bone, or that advised by Dr. Haygarth, which arrested the progress of an epidemic convulsion that occurred in North Wales, viz. by keeping those who were affected as separate as possible, and by preventing all girls and young women from having communication with each other,* would no doubt have been equally efficacious in the epidemic which has occurred in Cornwall, had it been attended to; but the majority of the spectators encouraged it from an idea that it was the operation of the Spirit of God.

*Falmouth, March* 18, 1814. JAMES CORNISH.

* See Haygarth on the Imagination, as a cause and as a cure of disorders of the body.

